# Supplementary material for: Cytotoxic Cytochalasans from Sponge-Derived Aspergillus sp. SCSIO 41044
Source: Molecules. 2025 Nov 20;30(22):4483. doi: 10.3390/molecules30224483 (PMC12655855; doi:10.3390/molecules30224483)
Supplement: Supplementary file 1 [file molecules-30-04483-s001.zip › molecules-3949119-supplementary.pdf]

Supporting Information

Cytotoxic Cytochalasans from Sponge-Derived *Aspergillus* sp. SCSIO 41044

Xiaoyan Pang [1,4,*], Ini Wong [3], Qinlin Cao [1,2], Junfeng Wang [1,4], Xuefeng Zhou [1], Bin Yang [1], Junjian Wang [3], Hong Wang [3,*] and Yonghong Liu [1,2,4,*]

[1] State Key Laboratory of Tropical Oceanography/Guangdong Key Laboratory of Marine Materia Medica, South China Sea Institute of Oceanology, Chinese Academy of Sciences, Guangzhou 510301, China
[2] University of Chinese Academy of Sciences, Beijing 100049, China
[3] Guangdong Province Engineering Laboratory for Druggability and New Drug Evaluation, School of Pharmaceutical Sciences, Sun Yat-sen University, Guangzhou 510006, China
[4] Sanya Institute of Marine Ecology and Engineering, Yazhou Scientific Bay, Sanya 572000, China
* Correspondence: xypang@scsio.ac.cn (X.P.); wangh759@mail.sysu.edu.cn (H.W.); yonghongliu@scsio.ac.cn (Y.L.)

List of Supporting Information

Figure S1. ^{1}H NMR spectrum of **1** in DMSO-d_6 at 500 MHz.
Figure S2. ^{13}C NMR spectrum of **1** in DMSO-d_6 at 125 MHz.
Figure S3. ^{1}H-^{1}H COSY spectrum of **1** in DMSO-d_6.
Figure S4. HSQC spectrum of **1** in DMSO-d_6.
Figure S5. HMBC spectrum of **1** in DMSO-d_6.
Figure S6. NOESY spectrum of **1** in DMSO-d_6.
Figure S7. HRESIMS spectrum of **1**.

Figure S8. ^{1}H NMR spectrum of **1a** in DMSO-d_6 at 700 MHz.
Figure S9. ^{1}H-^{1}H COSY spectrum of **1a** in DMSO-d_6.
Figure S10. ESIMS spectrum of **1a**.
Figure S11. ^{1}H NMR spectrum of **1b** in DMSO-d_6 at 700 MHz.
Figure S12. ^{1}H-^{1}H COSY spectrum of **1b** in DMSO-d_6.
Figure S13. ESIMS spectrum of **1b**.

Figure S14. ^{1}H NMR spectrum of **2** in DMSO-d_6 at 500 MHz.
Figure S15. ^{13}C NMR spectrum of **2** in DMSO-d_6 at 125 MHz.
Figure S16. ^{1}H-^{1}H COSY spectrum of **2** in DMSO-d_6.
Figure S17. HSQC spectrum of **2** in DMSO-d_6.
Figure S18. HMBC spectrum of **2** in DMSO-d_6.
Figure S19. HRESIMS spectrum of **2**.

Figure S20. ^{1}H NMR spectrum of **2a** in DMSO-d_6 at 500 MHz.
Figure S21. ^{1}H-^{1}H COSY spectrum of **2a** in DMSO-d_6.
Figure S22. ESIMS spectrum of **2a**.
Figure S23. ^{1}H NMR spectrum of **2b** in DMSO-d_6 at 500 MHz.
Figure S24. ^{1}H-^{1}H COSY spectrum of **2b** in DMSO-d_6.
Figure S25. ESIMS spectrum of **2b**.

Figure S26. ^{1}H NMR spectrum of **3** in DMSO-d_6 at 500 MHz.
Figure S27. ^{13}C NMR spectrum of **3** in DMSO-d_6 at 125 MHz.
Figure S28. ^{1}H-^{1}H COSY spectrum of **3** in DMSO-d_6.
Figure S29. HSQC spectrum of **3** in DMSO-d_6.
Figure S30. HMBC spectrum of **3** in DMSO-d_6.
Figure S31. NOESY spectrum of **3** in DMSO-d_6.
Figure S32. HRESIMS spectrum of **3**.
Table S1. NMR data for compounds **3** and **6** (500/125 MHz, TMS, δ ppm) in DMSO-d_6.
Figure S33. Key ^{1}H-^{1}H COSY (—), HMBC (→), and NOESY (↔) correlations of compound **3**.
ITS1-5.8S-ITS2 sequences of *Aspergillus* sp. SCSIO 41044

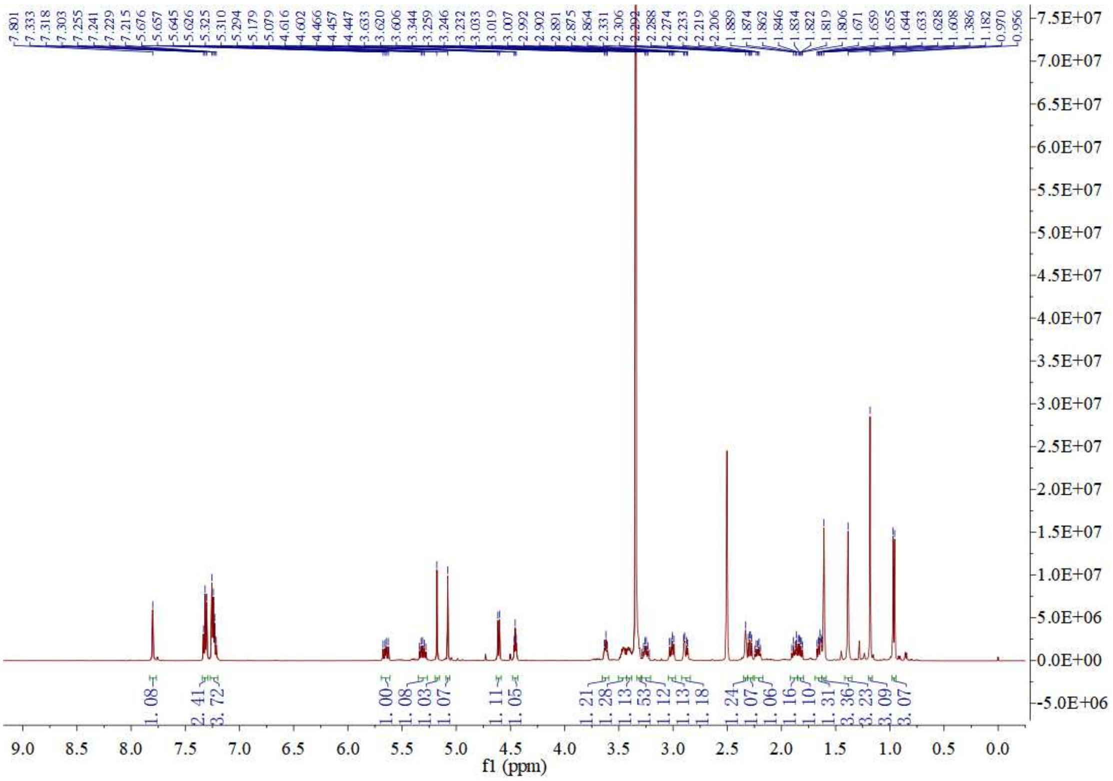

Figure S1. ^{1}H NMR spectrum of **1** in DMSO-d_6 at 500 MHz.

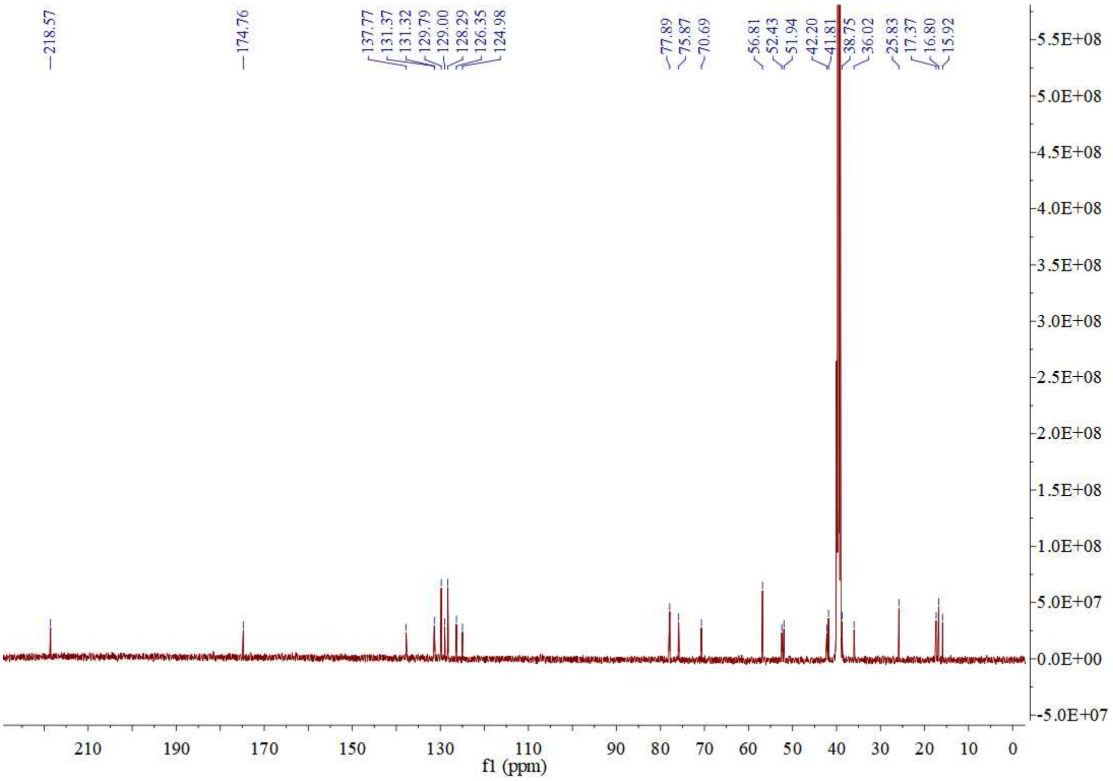

Figure S2. ^{13}C NMR spectrum of **1** in DMSO-d_6 at 125 MHz.

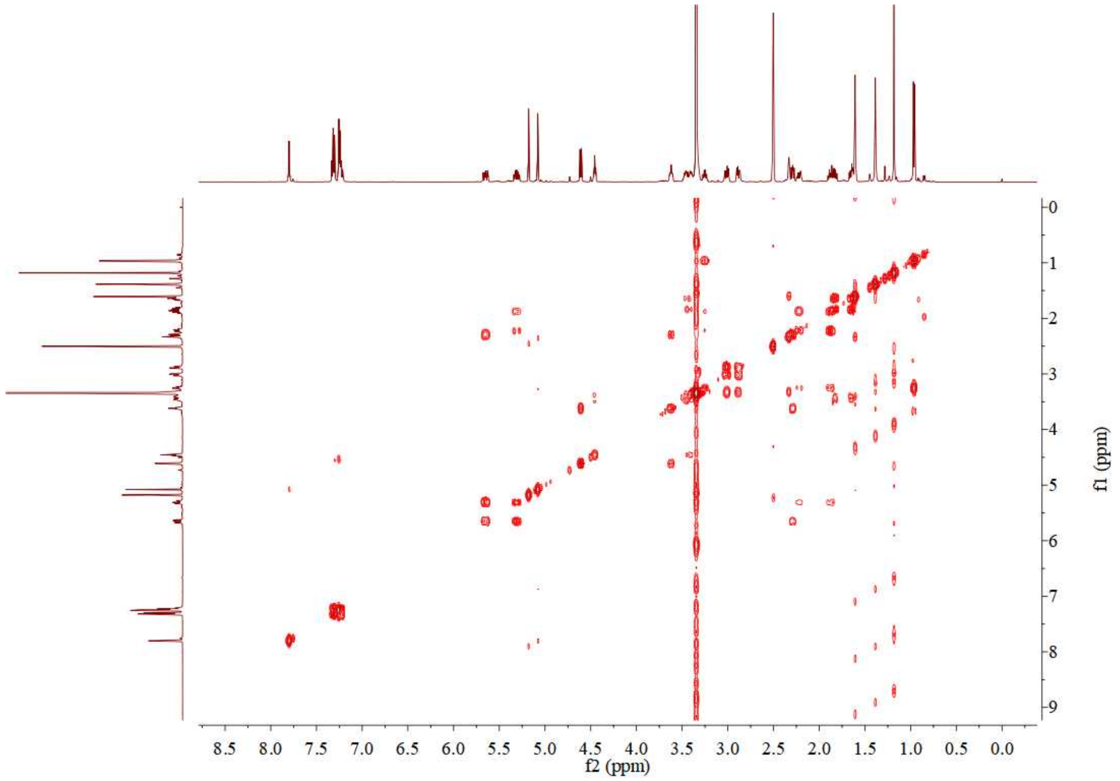

Figure S3. ^{1}H-^{1}H COSY spectrum of **1** in DMSO-d_6.

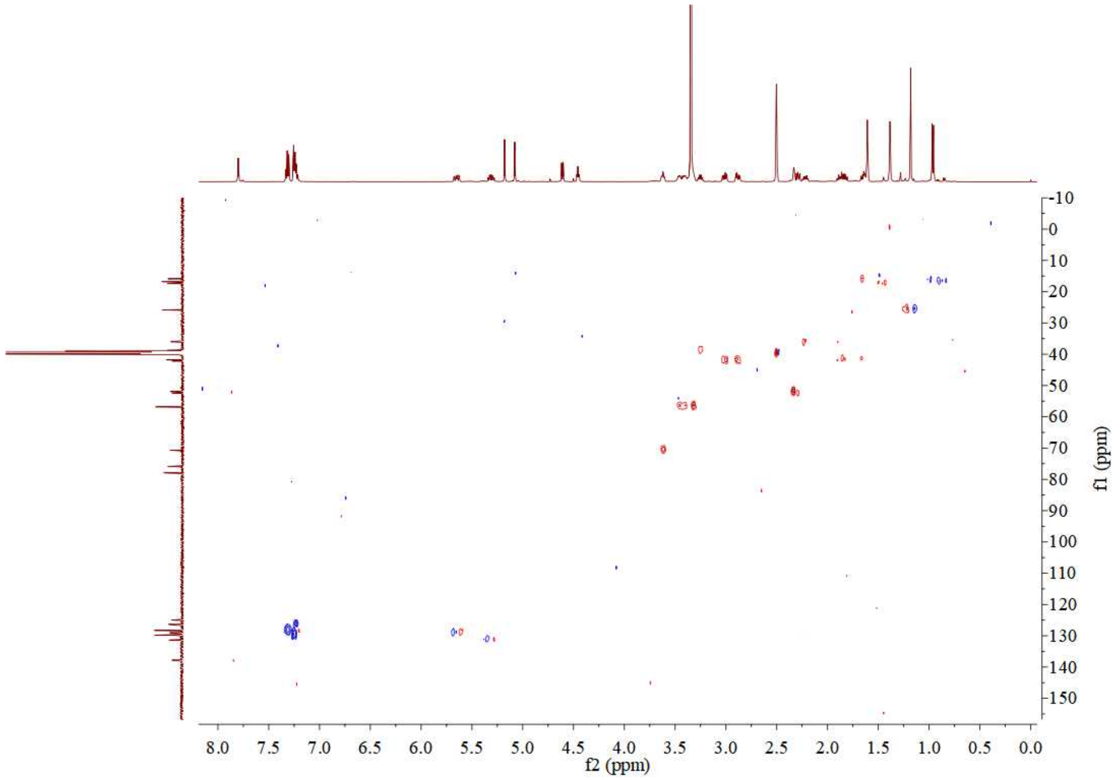

Figure S4. HSQC spectrum of **1** in DMSO-d_6.

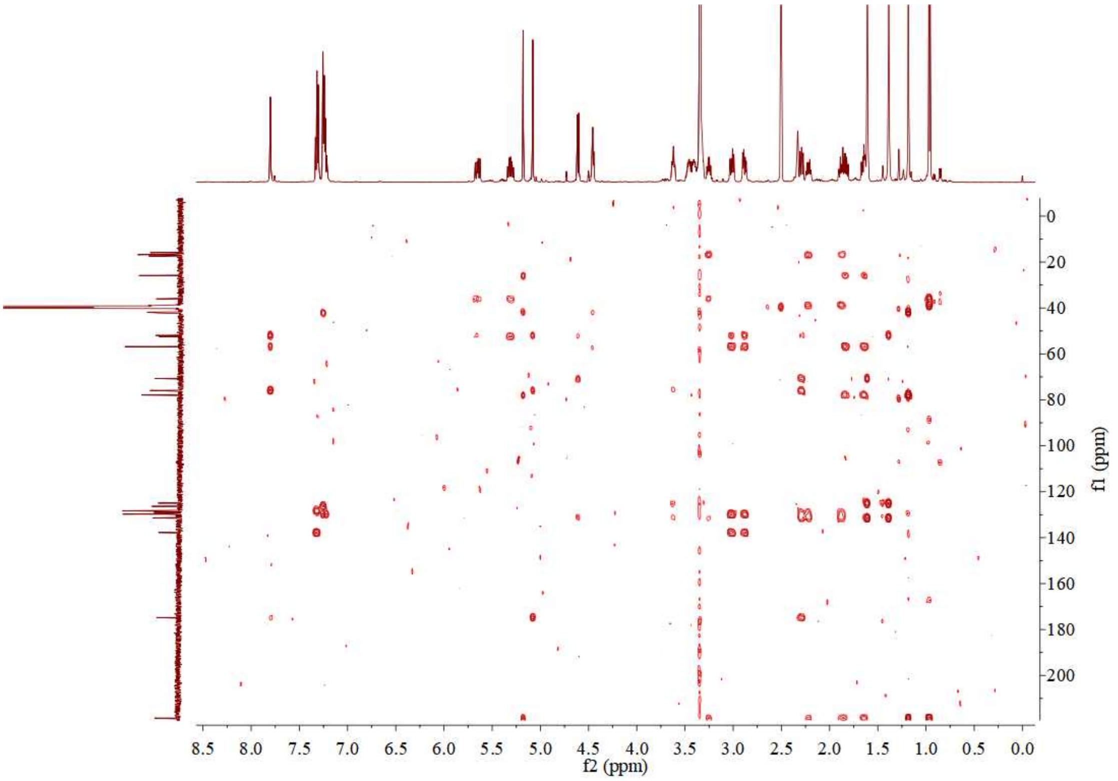

Figure S5. HMBC spectrum of **1** in DMSO-d_6.

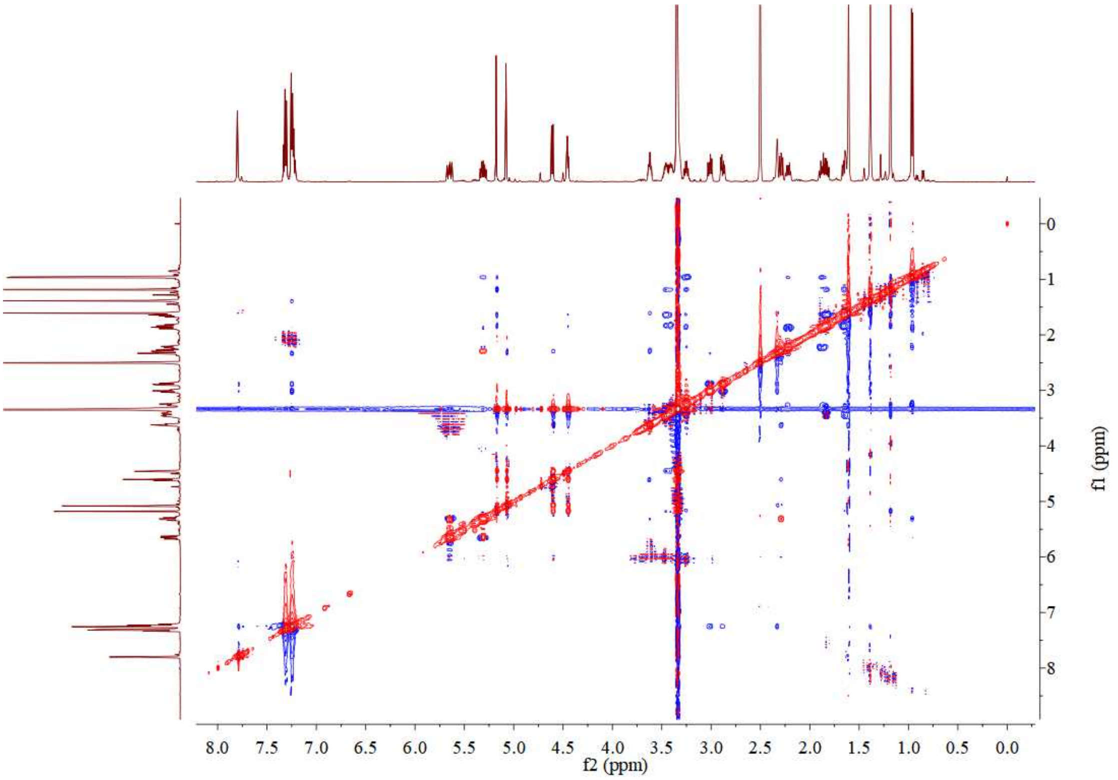

Figure S6. NOESY spectrum of **1** in DMSO-d_6.

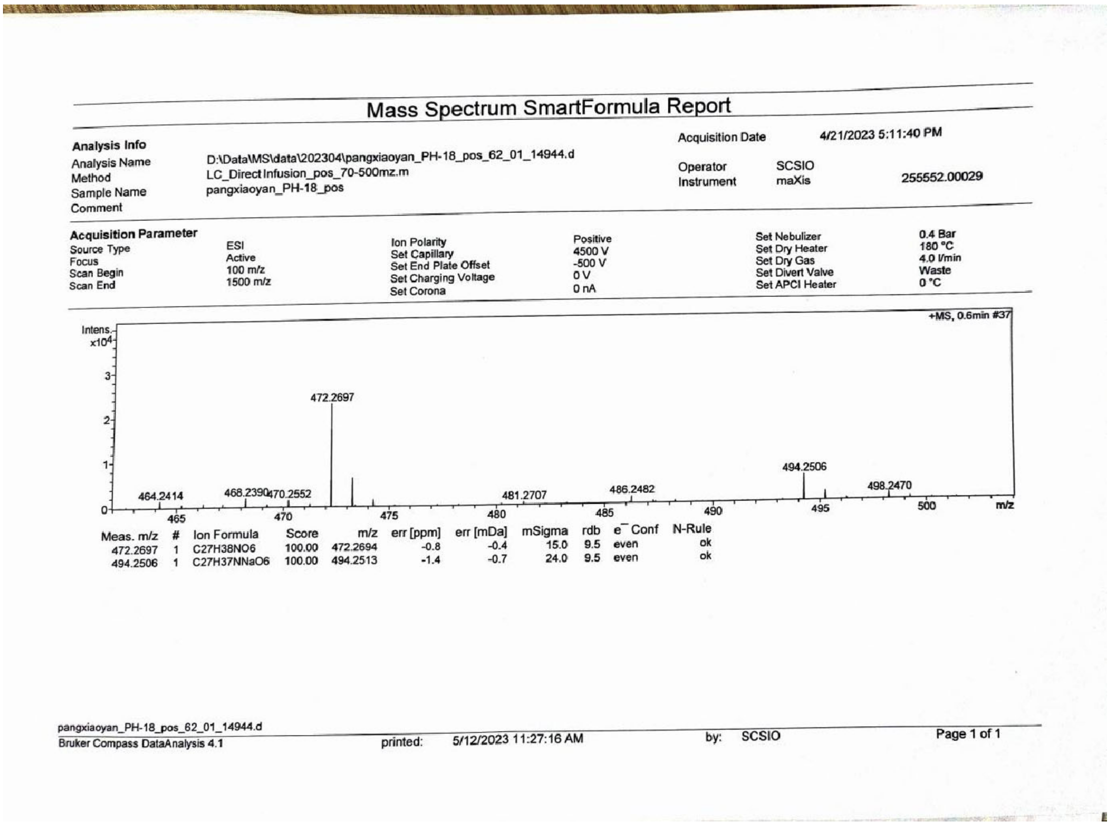

Figure S7. HRESIMS spectrum of **1**.

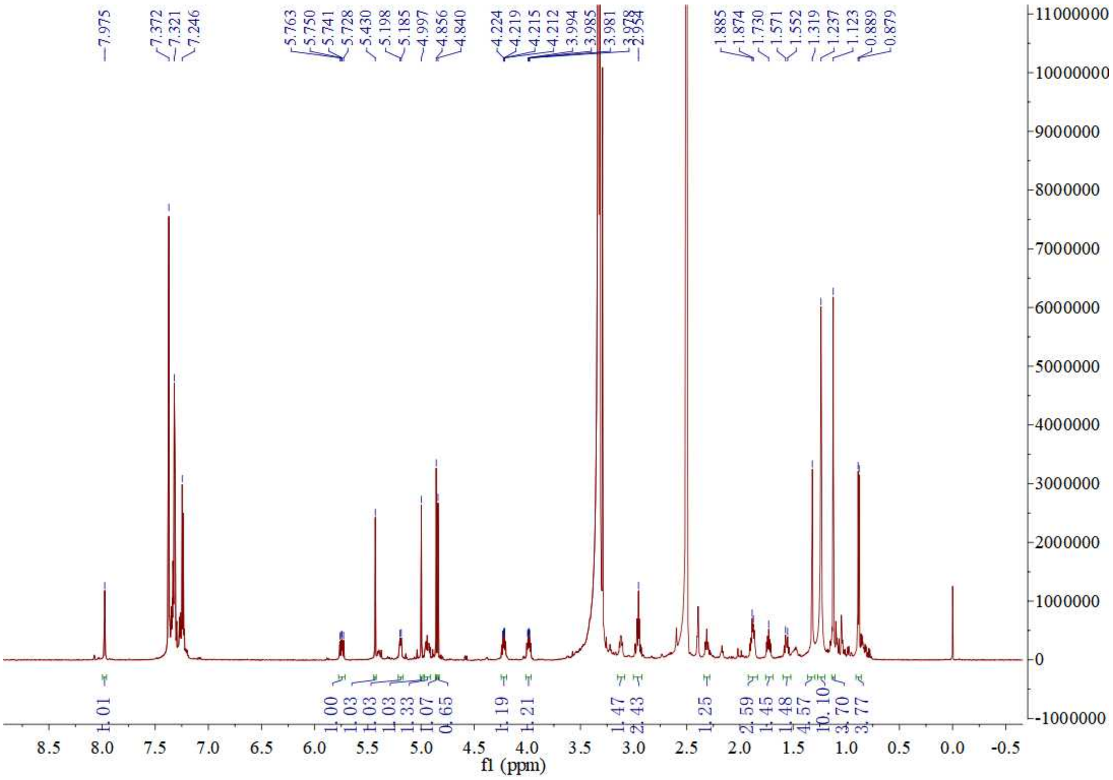

Figure S8. ^{1}H NMR spectrum of **1a** in DMSO-d_6 at 700 MHz.

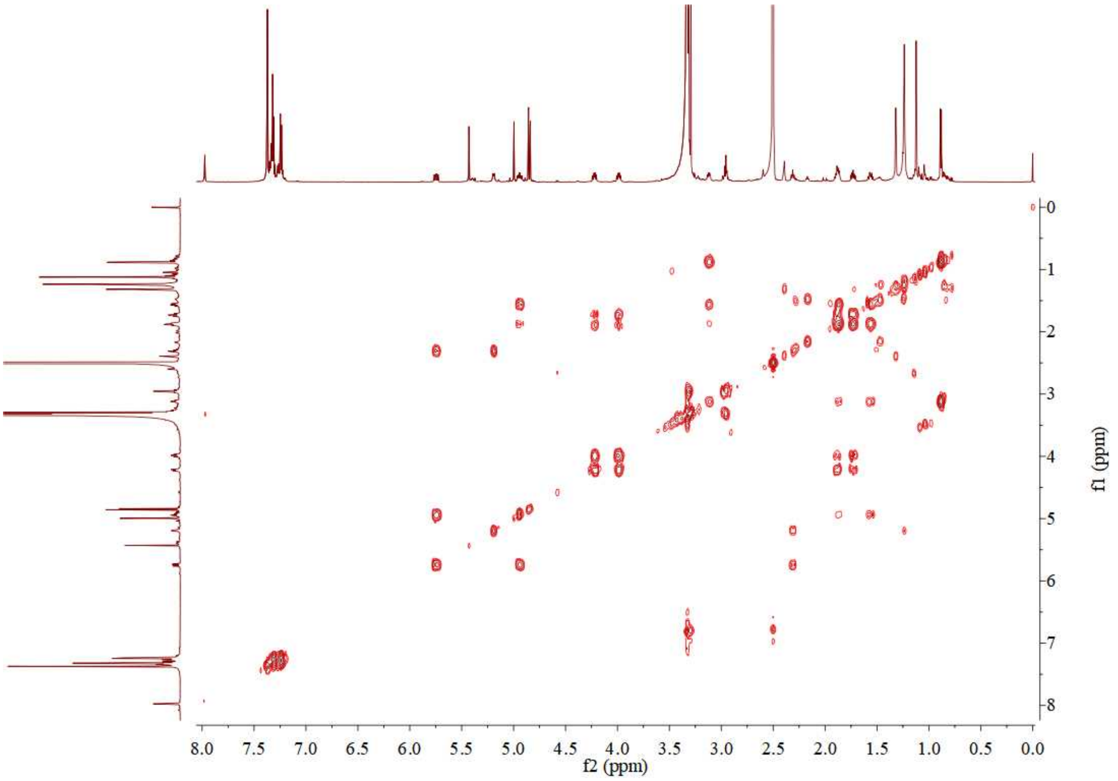

Figure S9. ^{1}H-^{1}H COSY spectrum of **1a** in DMSO-d_6.

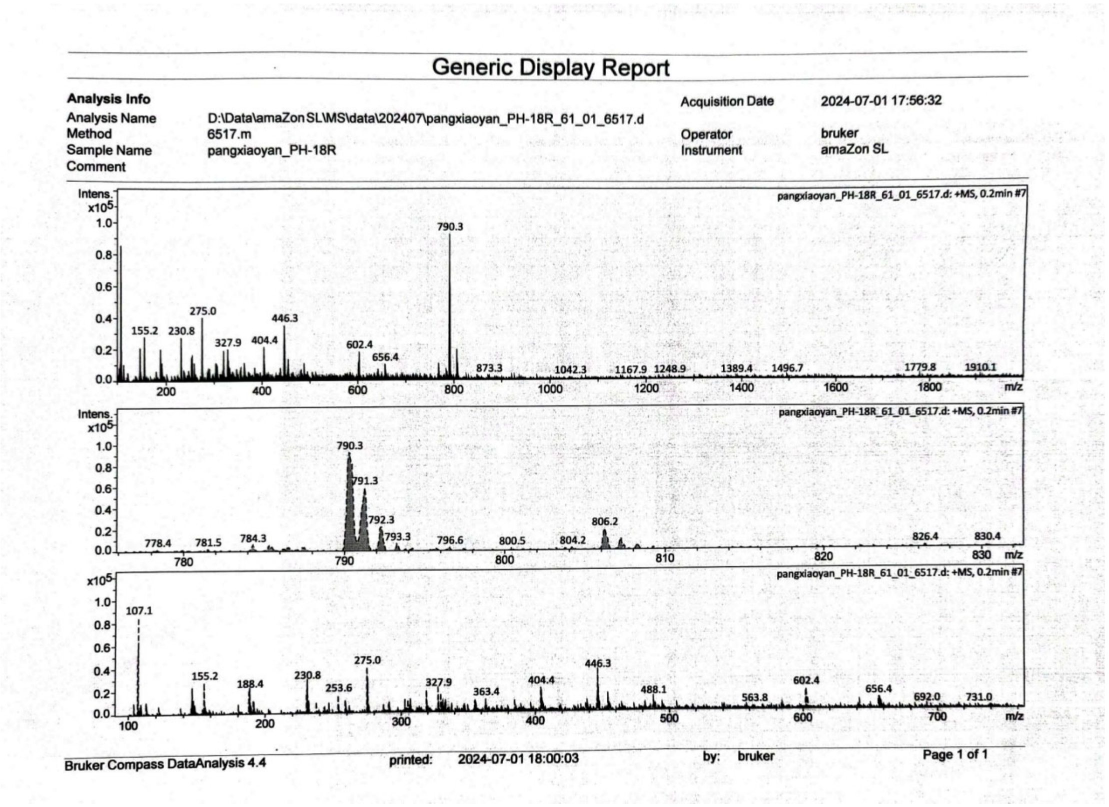

Figure S10. ESIMS spectrum of **1a**.

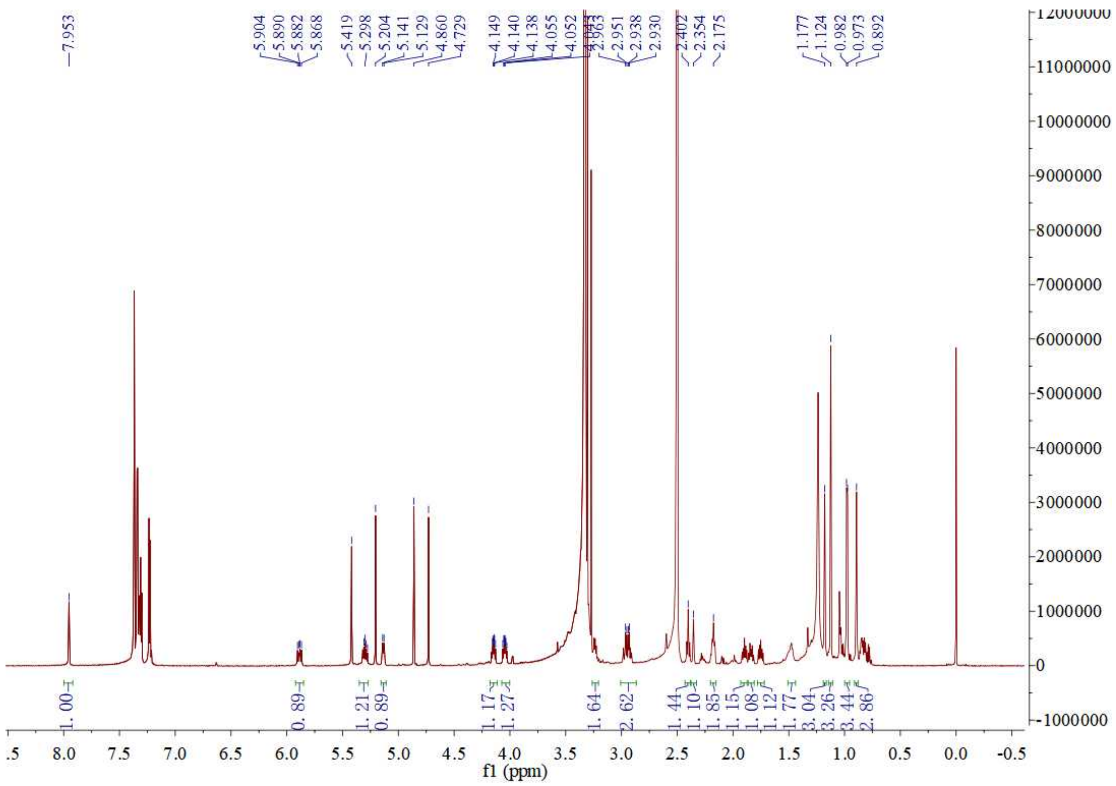

Figure S11. ^{1}H NMR spectrum of **1b** in DMSO-d_6 at 700 MHz.

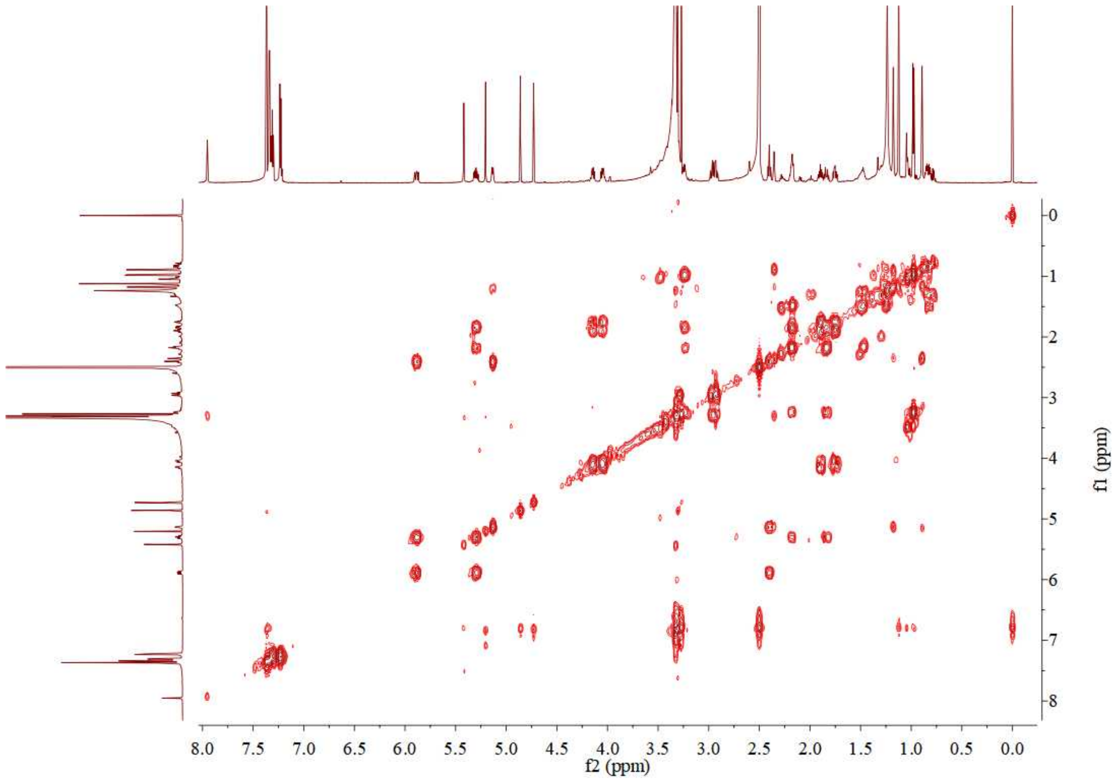

Figure S12. ^{1}H-^{1}H COSY spectrum of **1b** in DMSO-d_6.

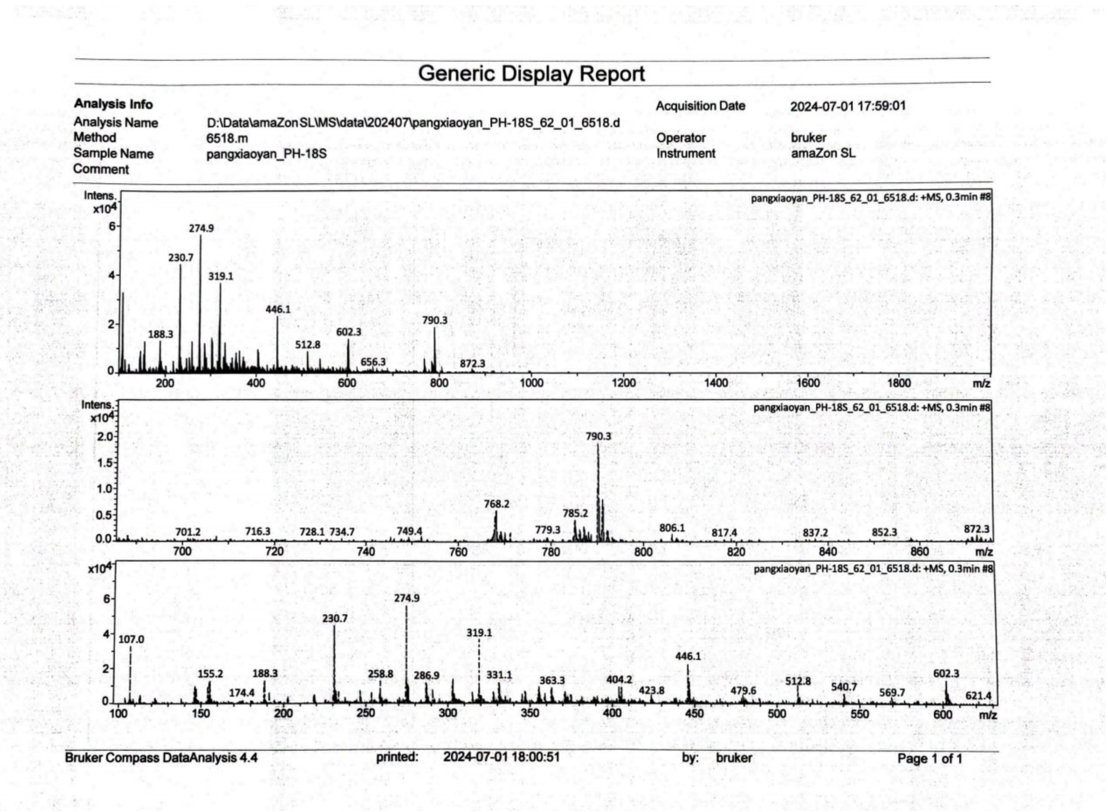

Figure S13. ESIMS spectrum of **1b**.

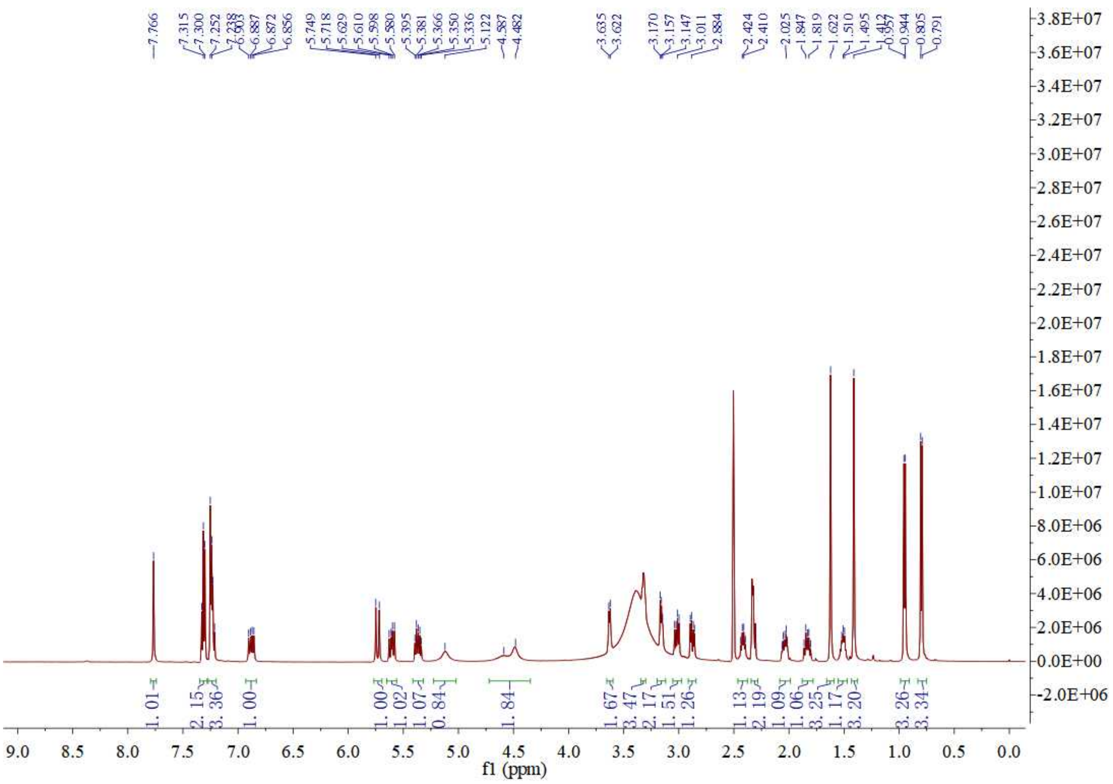

Figure S14. ^{1}H NMR spectrum of **2** in DMSO-d_6 at 500 MHz.

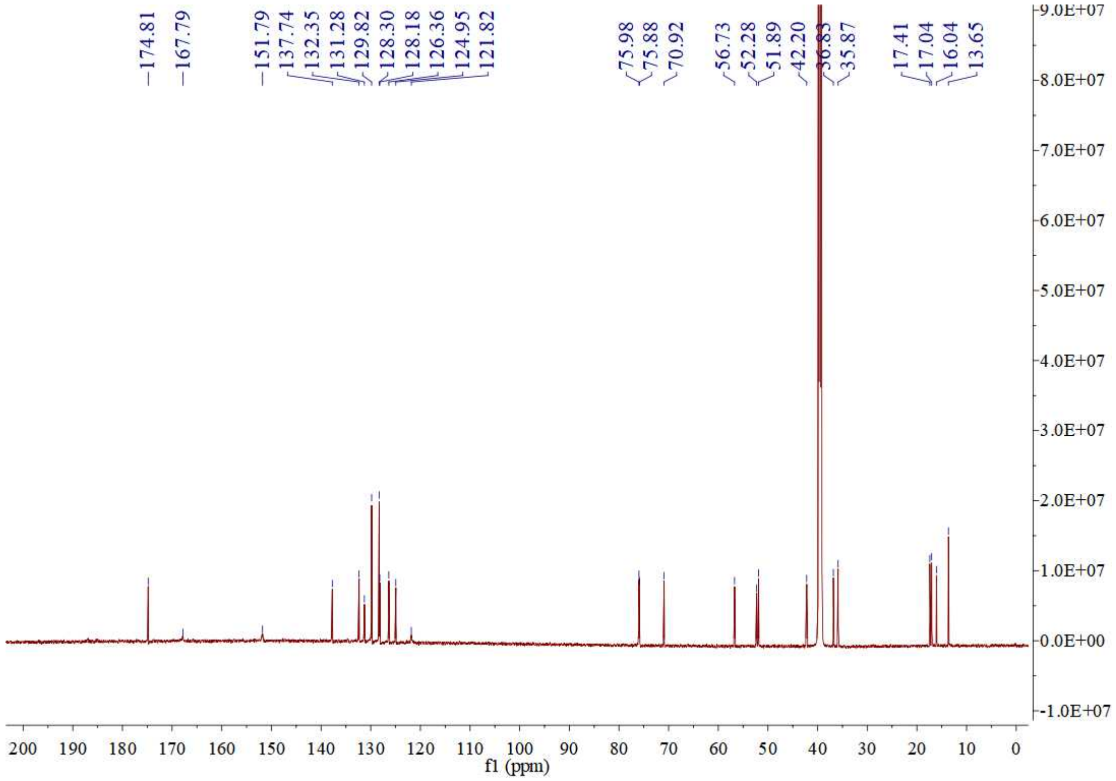

Figure S15. ^{13}C NMR spectrum of **2** in DMSO-d_6 at 125 MHz.

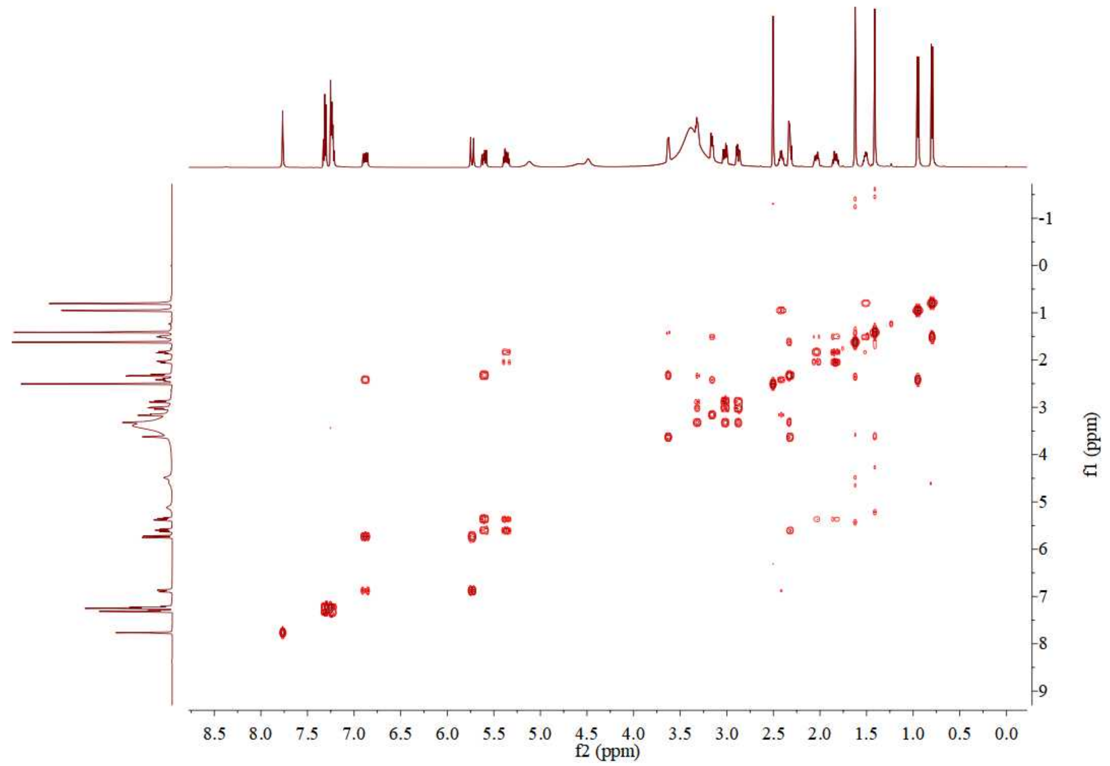

Figure S16. ^{1}H-^{1}H COSY spectrum of **2** in DMSO-d_6.

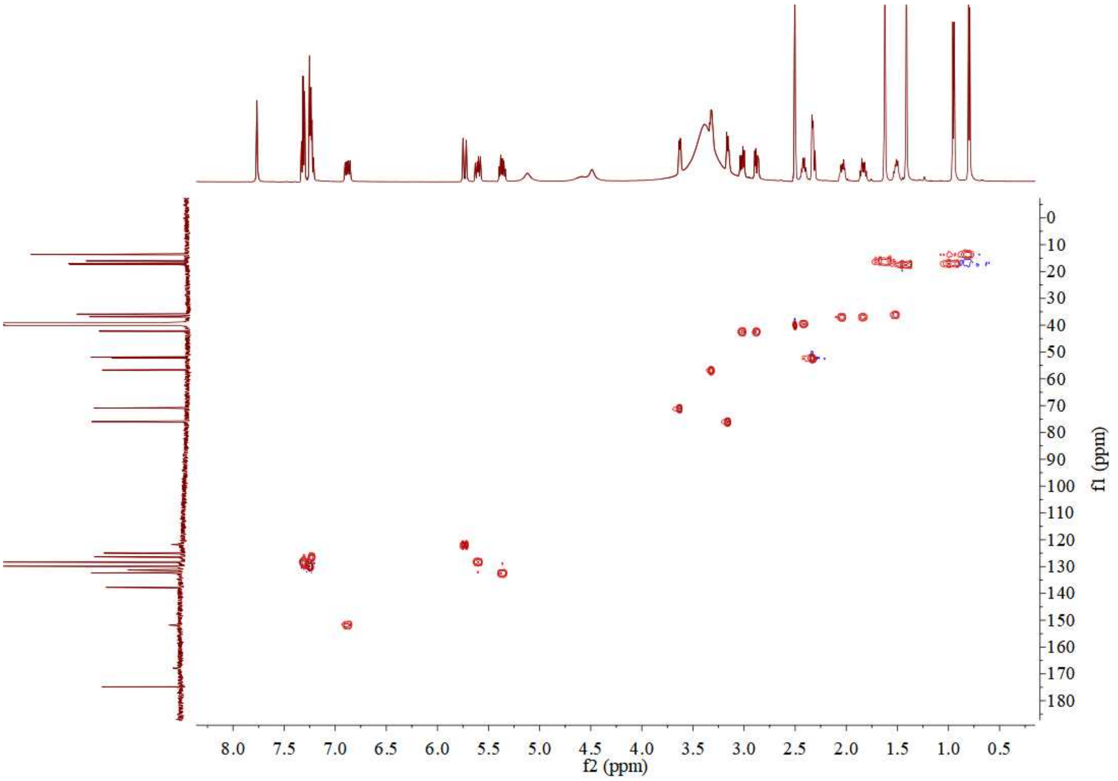

Figure S17. HSQC spectrum of **2** in DMSO-d_6.

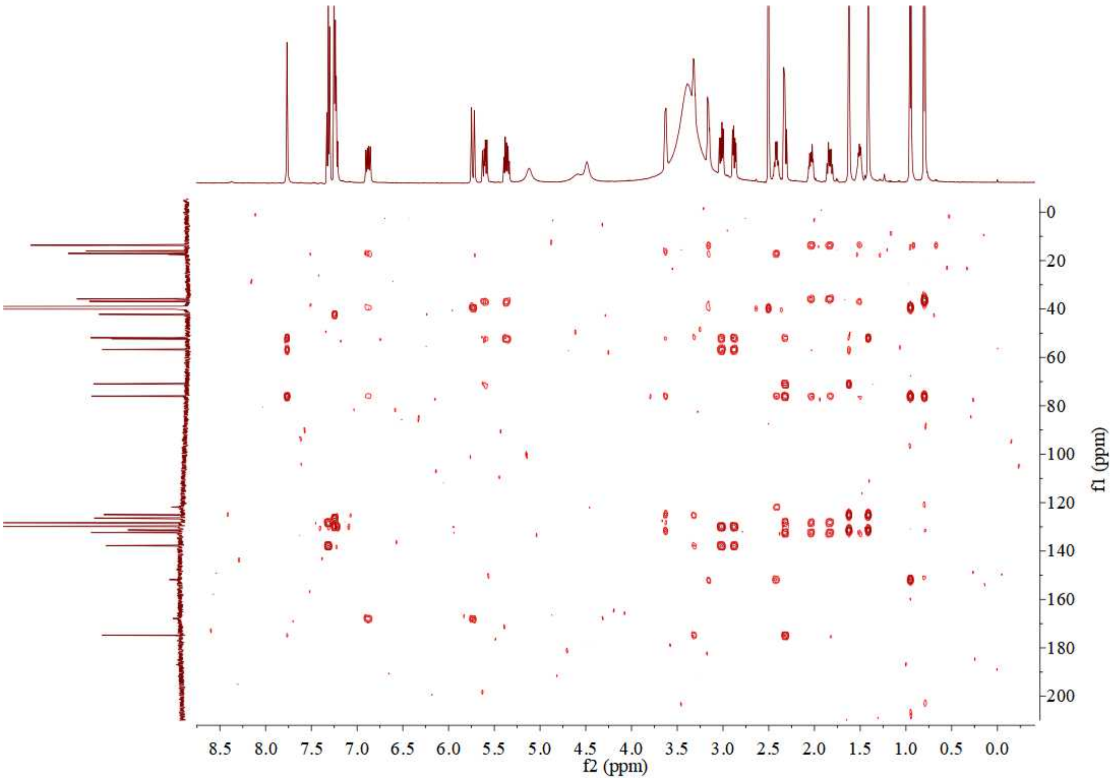

Figure S18. HMBC spectrum of **2** in DMSO-d_6.

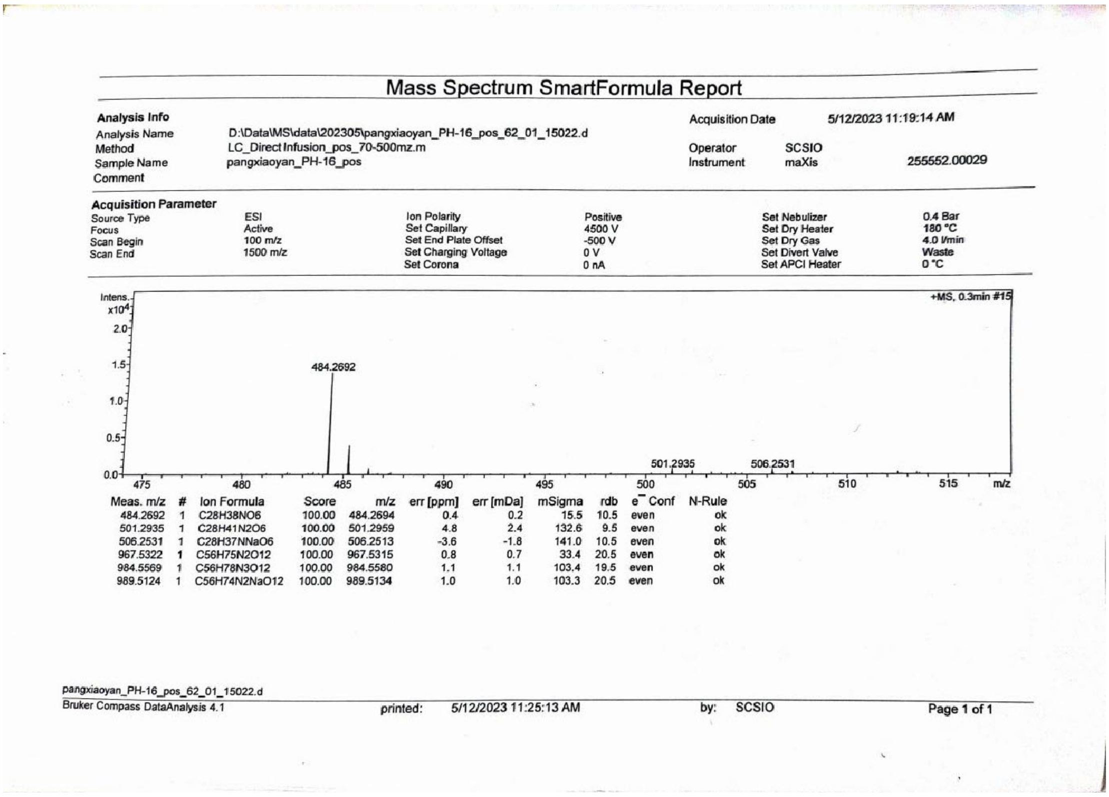

Mass Spectrum SmartFormula Report

Analysis Info

Analysis Name	D:\Data\MS\data\202305\pangxiaoyan_PH-16_pos_62_01_15022.d	Acquisition Date	5/12/2023 11:19:14 AM
Method	LC_Direct Infusion_pos_70-500mz.m	Operator	SCSIO
Sample Name	pangxiaoyan_PH-16_pos	Instrument	maXis 255552.00029
Comment			

Acquisition Parameter

Source Type	ESI	Ion Polarity	Positive	Set Nebulizer	0.4 Bar
Focus	Active	Set Capillary	4500 V	Set Dry Heater	180 °C
Scan Begin	100 m/z	Set End Plate Offset	-500 V	Set Dry Gas	4.0 l/min
Scan End	1500 m/z	Set Charging Voltage	0 V	Set Divert Valve	Waste
		Set Corona	0 nA	Set APCI Heater	0 °C

Meas. m/z	#	Ion Formula	Score	m/z	err [ppm]	err [mDa]	mSigma	rdb	e⁻ Conf	N-Rule
484.2692	1	C28H38NO6	100.00	484.2694	0.4	0.2	15.5	10.5	even	ok
501.2935	1	C28H41N2O6	100.00	501.2959	4.8	2.4	132.6	9.5	even	ok
506.2531	1	C28H37NNaO6	100.00	506.2513	-3.6	-1.8	141.0	10.5	even	ok
967.5322	1	C56H75N2O12	100.00	967.5315	0.8	0.7	33.4	20.5	even	ok
984.5569	1	C56H78N3O12	100.00	984.5580	1.1	1.1	103.4	19.5	even	ok
989.5124	1	C56H74N2NaO12	100.00	989.5134	1.0	1.0	103.3	20.5	even	ok

pangxiaoyan_PH-16_pos_62_01_15022.d

Bruker Compass DataAnalysis 4.1 printed: 5/12/2023 11:25:13 AM by: SCSIO Page 1 of 1

Figure S19. HRESIMS spectrum of **2**.

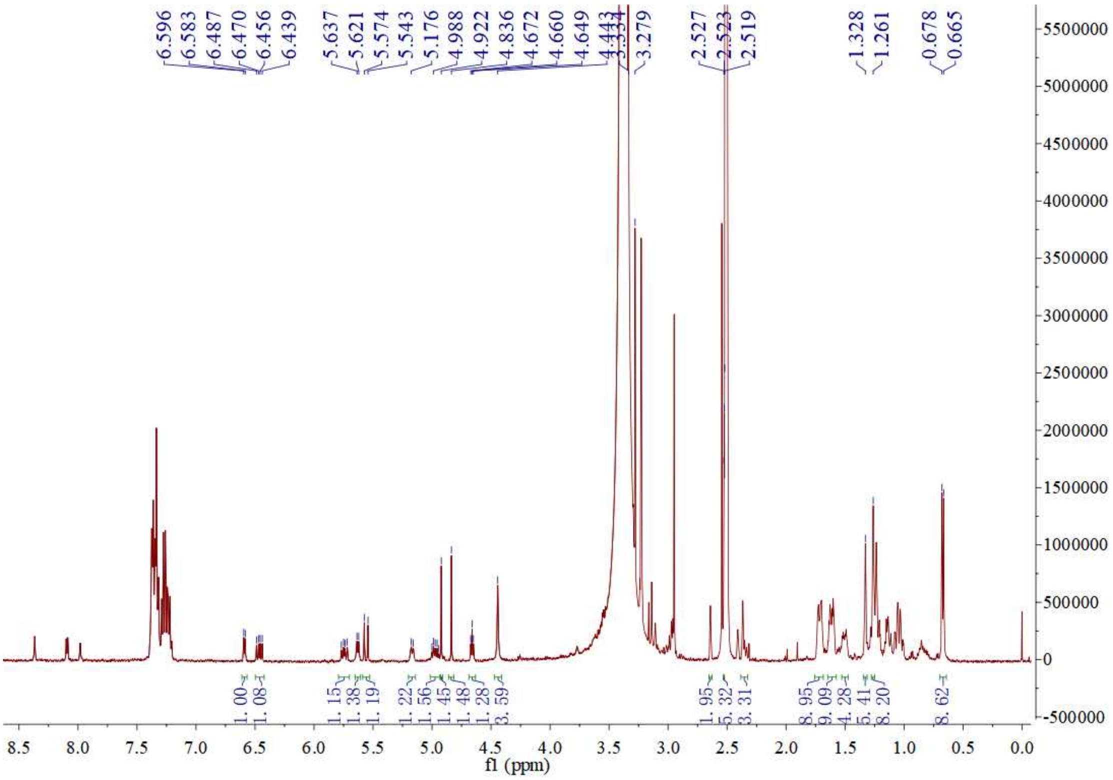

Figure S20. ^{1}H NMR spectrum of **2a** in DMSO-d_6 at 500 MHz.

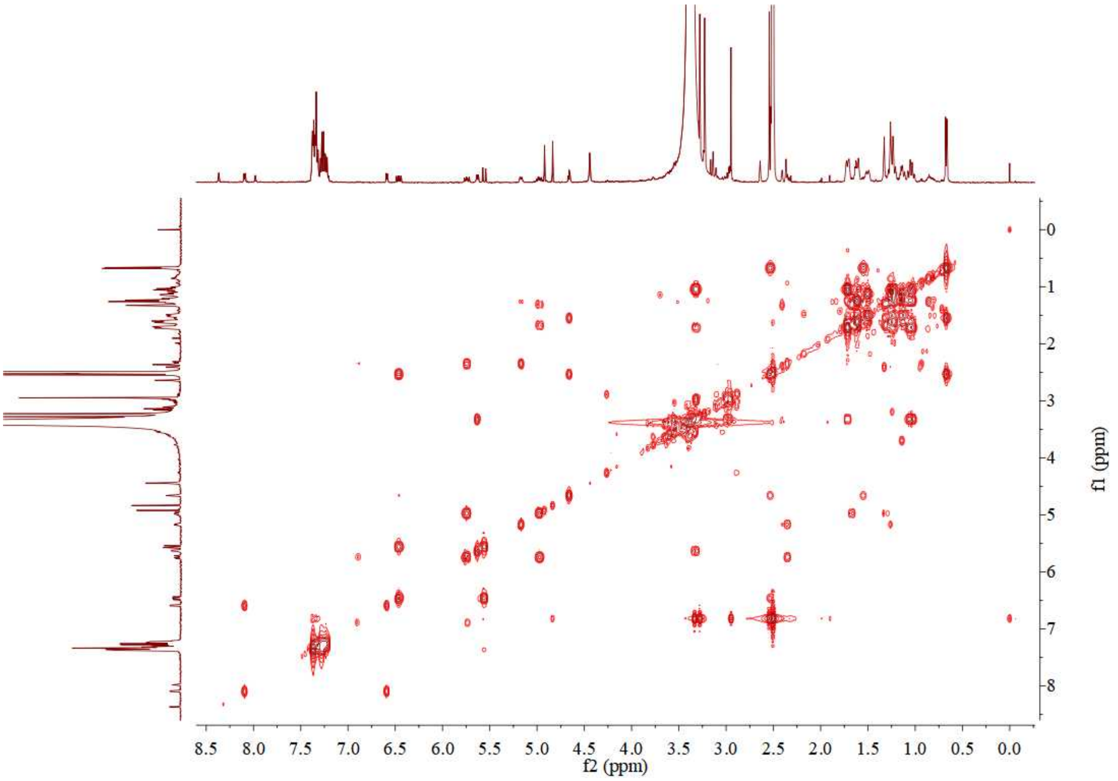

Figure S21. ^{1}H-^{1}H COSY spectrum of **2a** in DMSO-d_6.

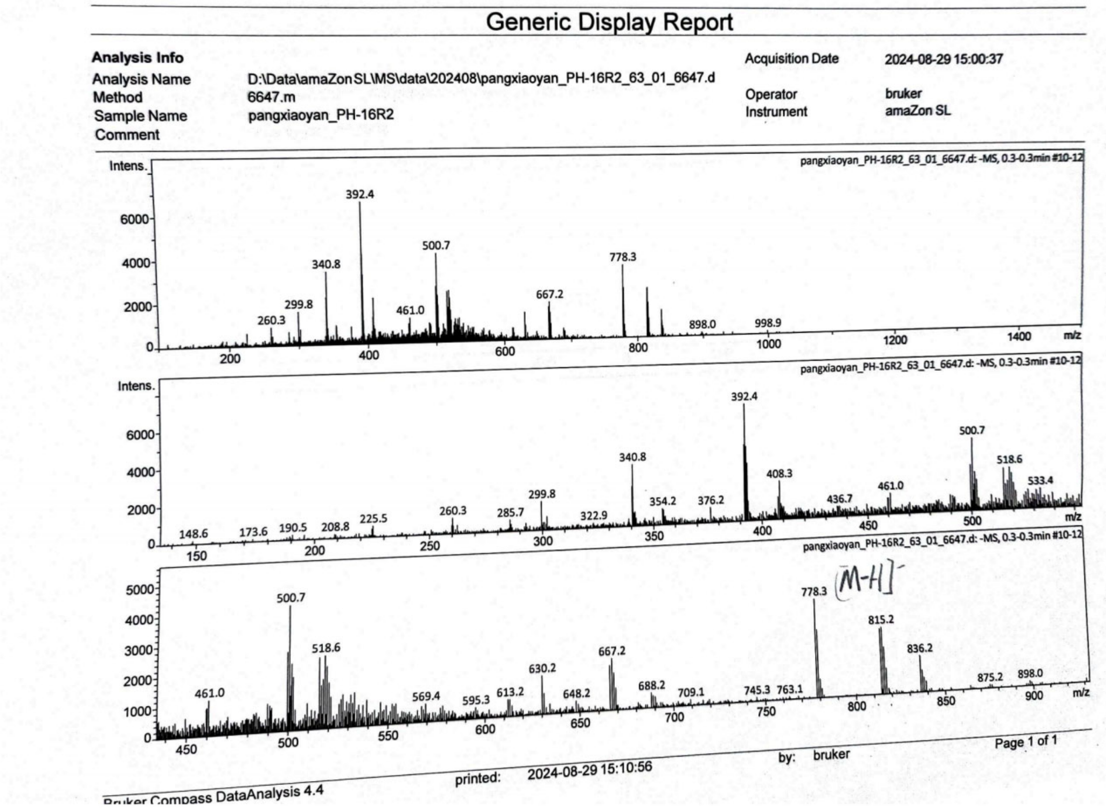

Figure S22. ESIMS spectrum of **2a**.

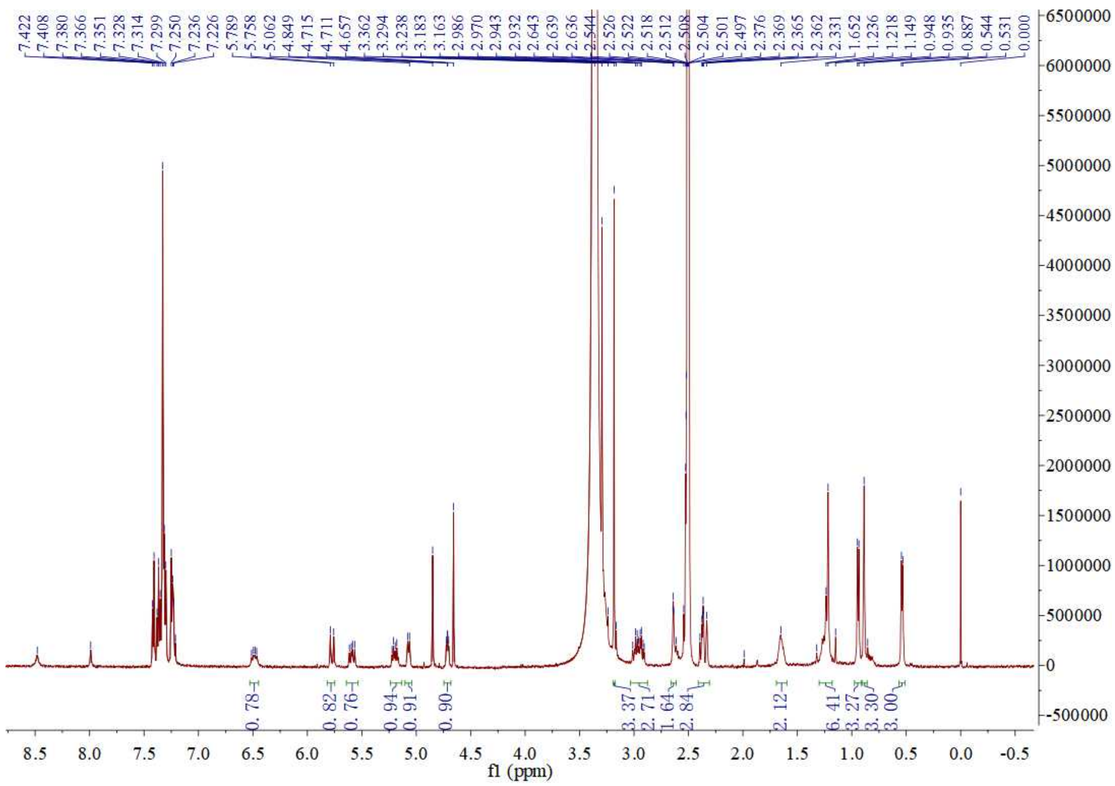

Figure S23. ^{1}H NMR spectrum of **2b** in DMSO-d_6 at 500 MHz.

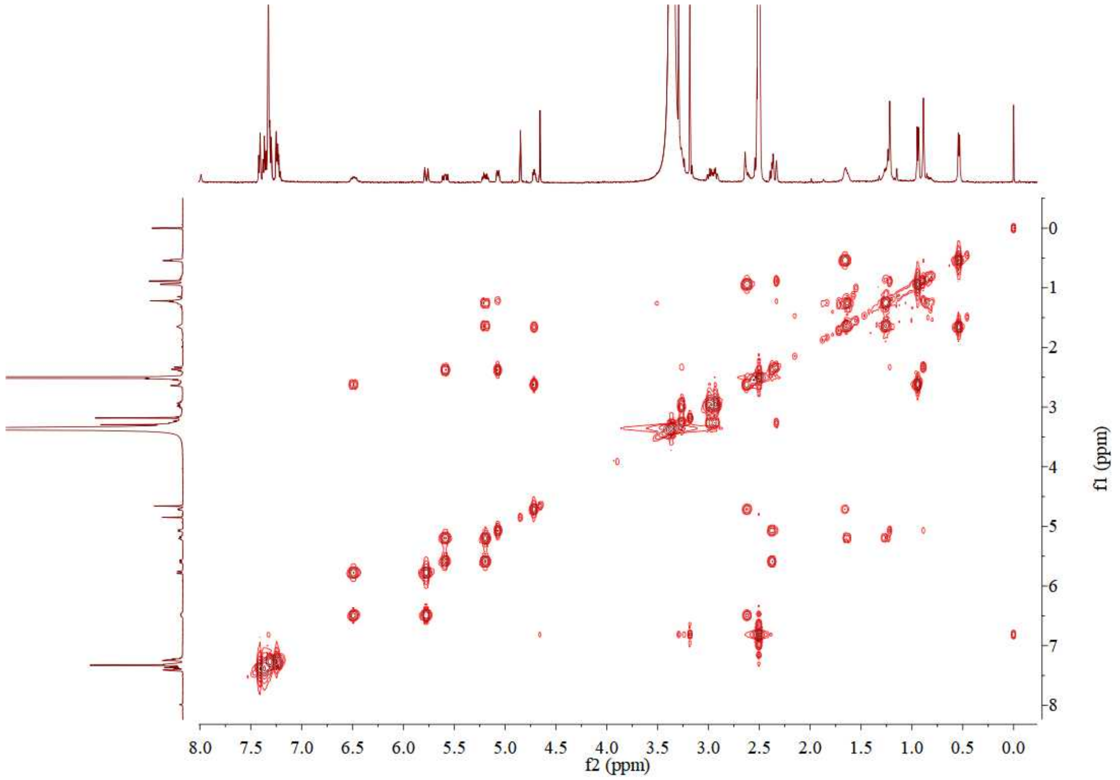

Figure S24. ^{1}H-^{1}H COSY spectrum of **2b** in DMSO-d_6.

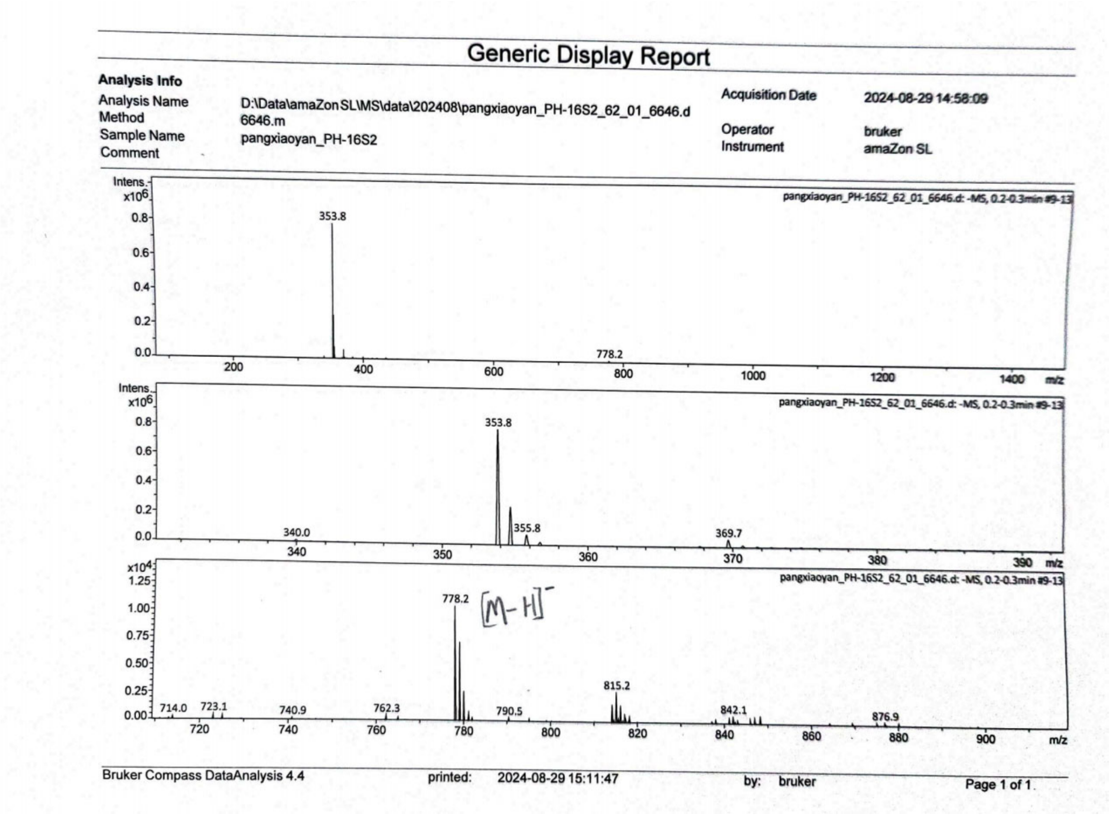

Figure S25. ESIMS spectrum of **2b**.

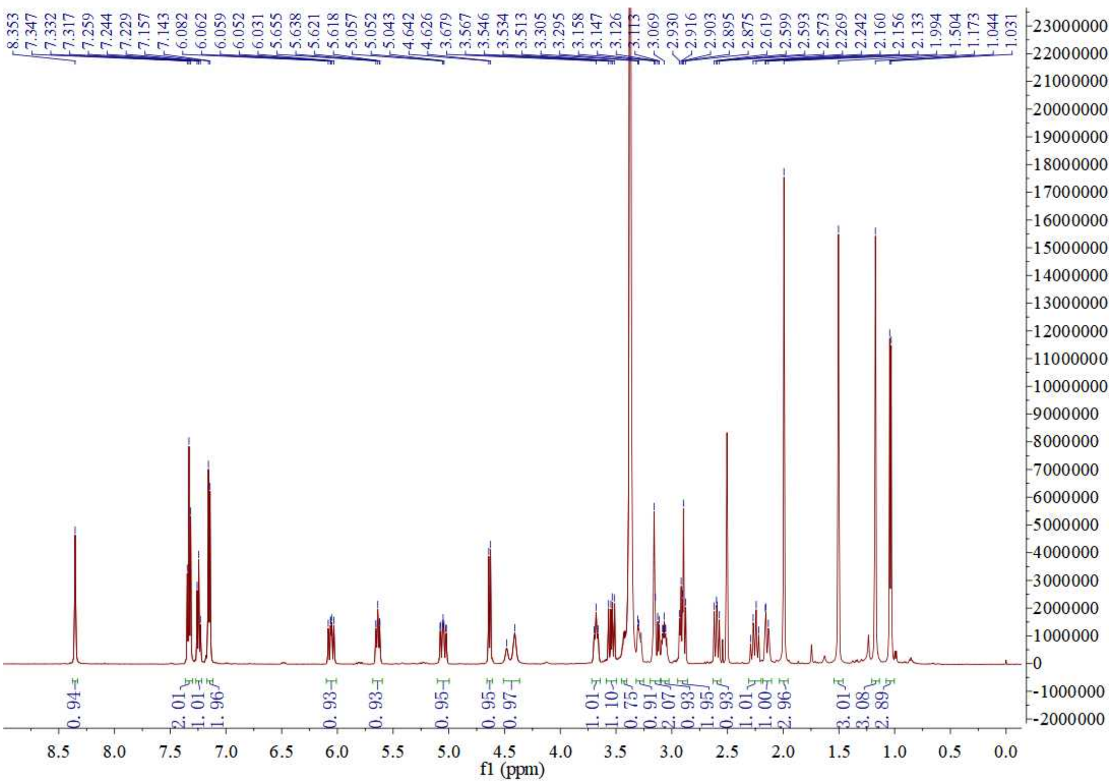

Figure S26. ^{1}H NMR spectrum of **3** in DMSO-d_6 at 500 MHz.

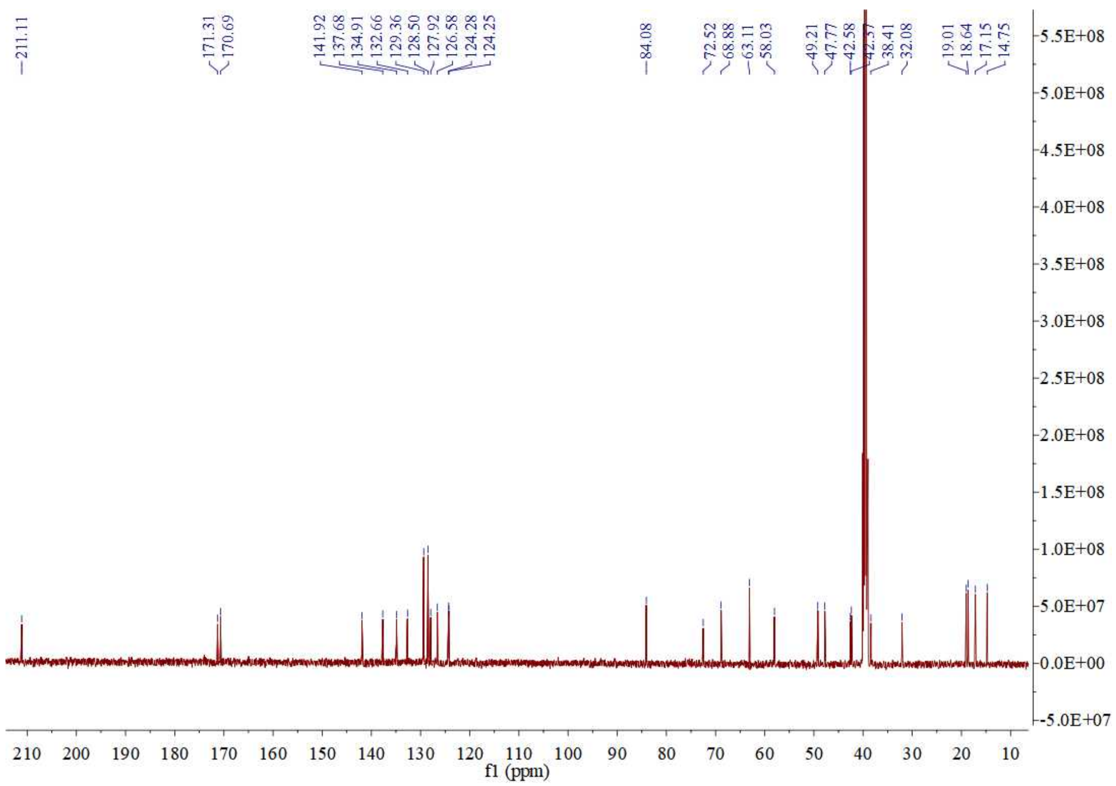

Figure S27. ^{13}C NMR spectrum of **3** in DMSO-d_6 at 125 MHz.

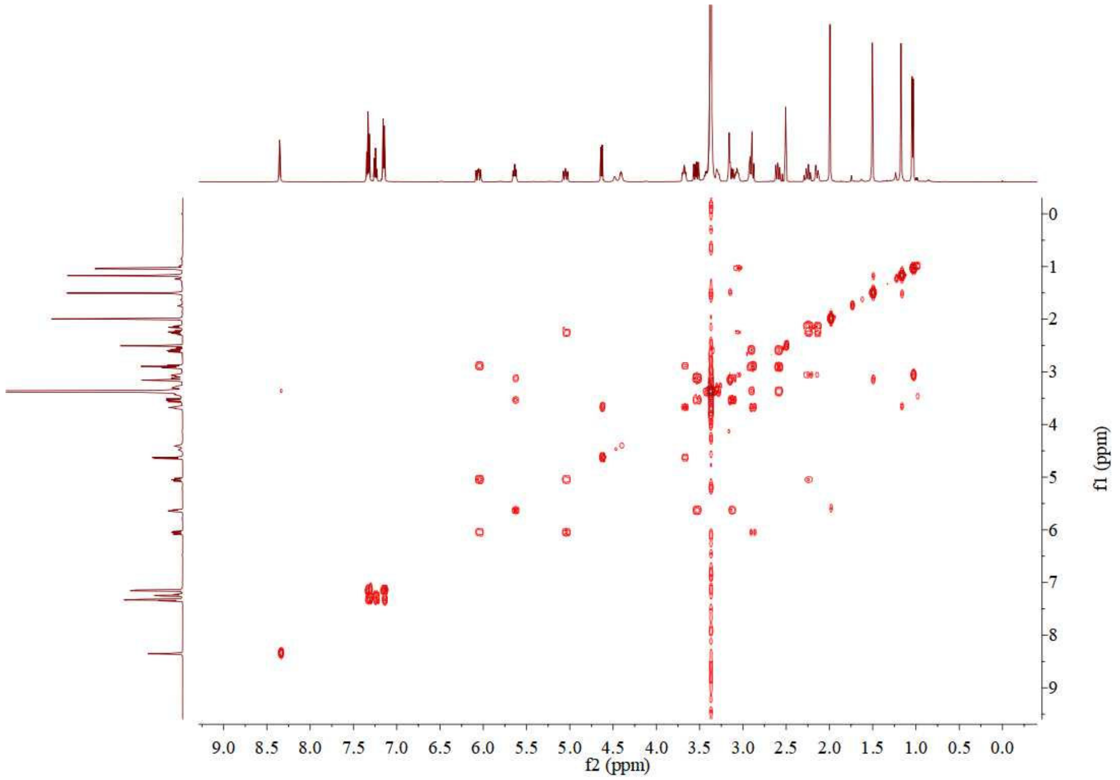

Figure S28. ^{1}H-^{1}H COSY spectrum of **3** in DMSO-d_6.

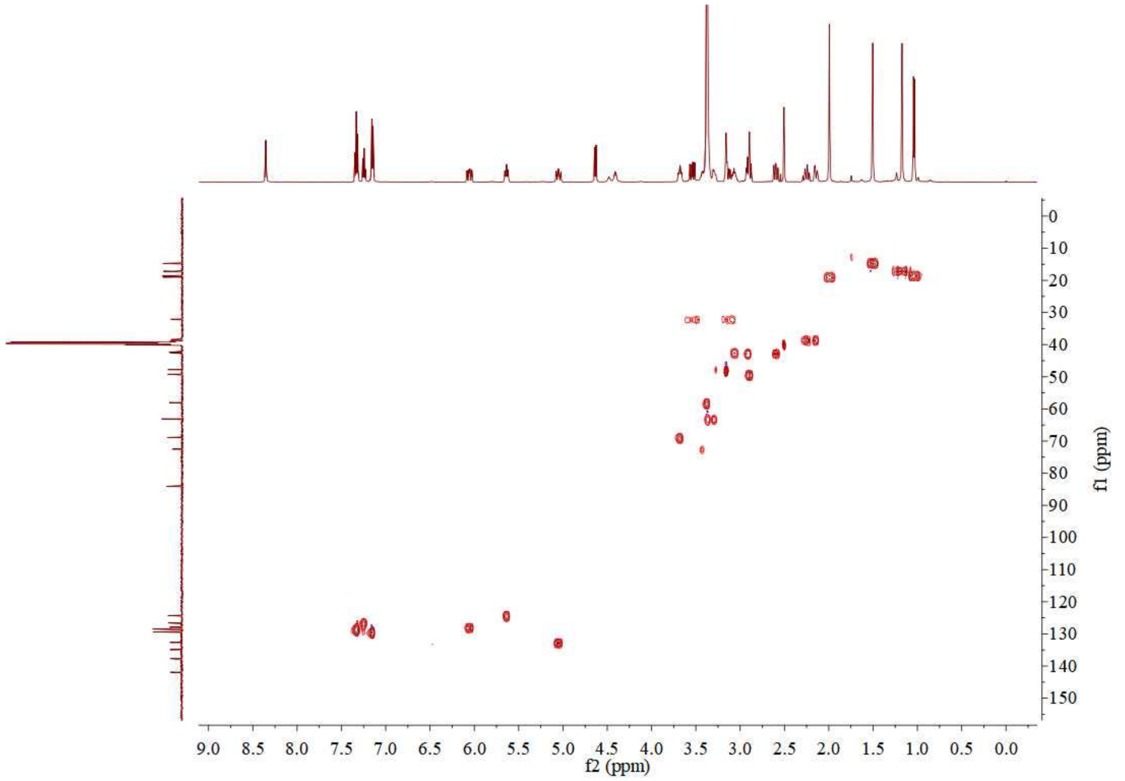

Figure S29. HSQC spectrum of **3** in DMSO-d_6.

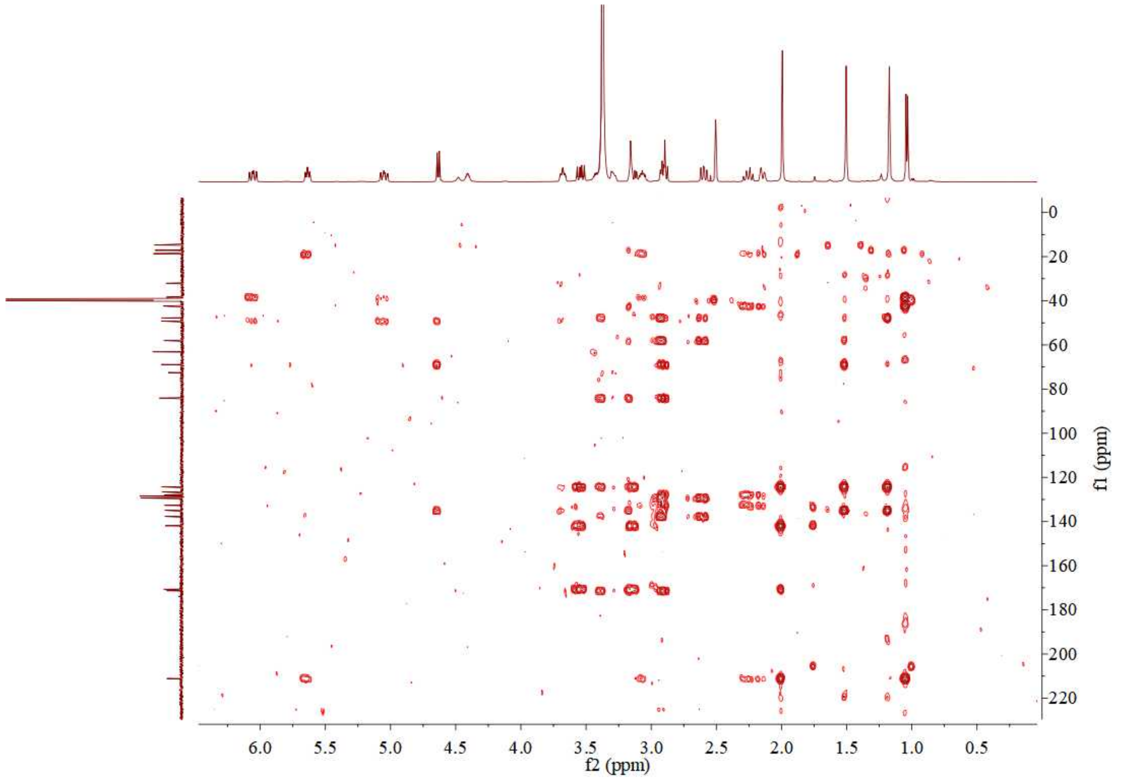

Figure S30. HMBC spectrum of **3** in DMSO-d_6.

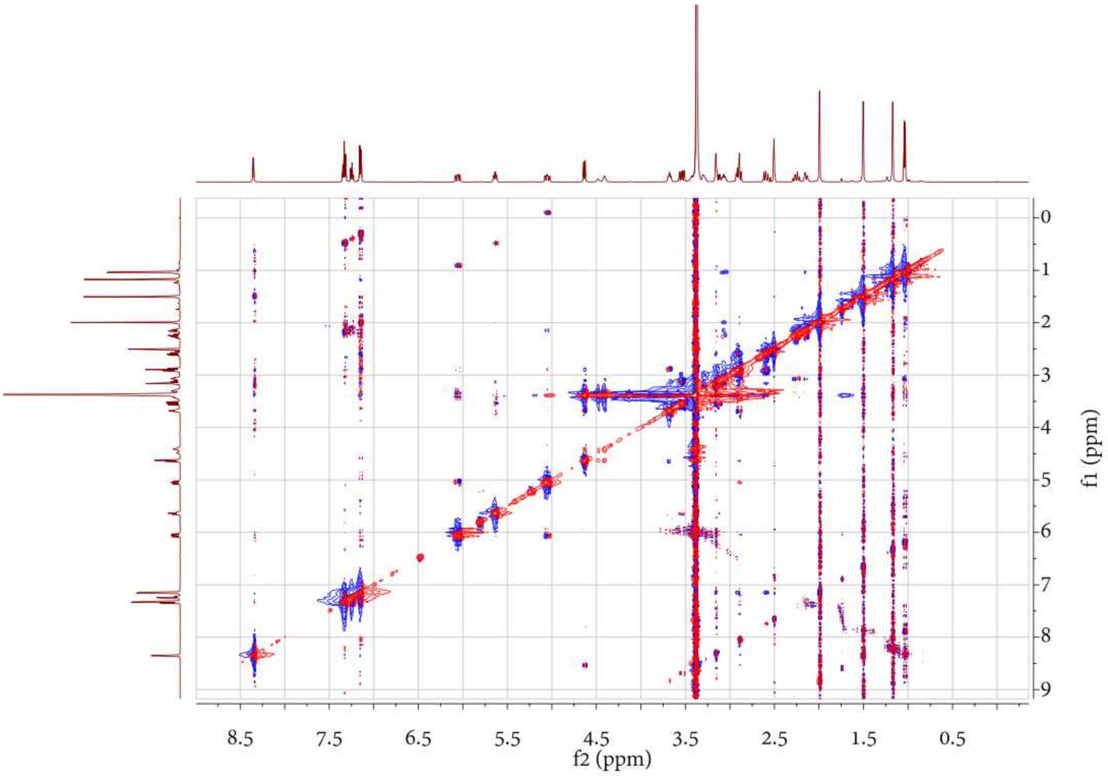

Figure S31. NOESY spectrum of **3** in DMSO-d_6.

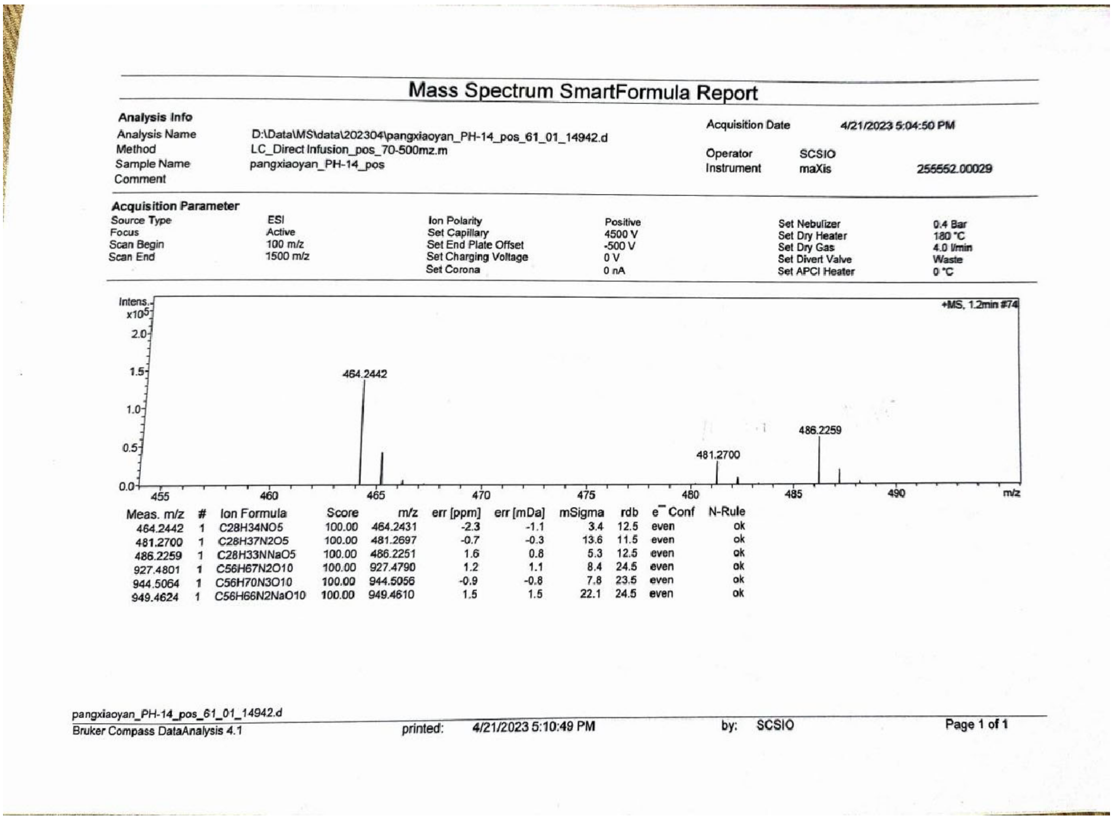

Mass Spectrum SmartFormula Report

Analysis Info

Analysis Name	D:\Data\MS\data\202304\pangxiaoyan_PH-14_pos_61_01_14942.d	Acquisition Date	4/21/2023 5:04:50 PM
Method	LC_Direct Infusion_pos_70-500mz.m	Operator	SCSIO
Sample Name	pangxiaoyan_PH-14_pos	Instrument	maXis 255552.00029
Comment			

Acquisition Parameter

Source Type	ESI	Ion Polarity	Positive	Set Nebulizer	0.4 Bar
Focus	Active	Set Capillary	4500 V	Set Dry Heater	180 °C
Scan Begin	100 m/z	Set End Plate Offset	-500 V	Set Dry Gas	4.0 l/min
Scan End	1500 m/z	Set Charging Voltage	0 V	Set Divert Valve	Waste
		Set Corona	0 nA	Set APCI Heater	0 °C

Meas. m/z	#	Ion Formula	Score	m/z	err [ppm]	err [mDa]	mSigma	rdb	e⁻ Conf	N-Rule
464.2442	1	C28H34NO5	100.00	464.2431	-2.3	-1.1	3.4	12.5	even	ok
481.2700	1	C28H37N2O5	100.00	481.2697	-0.7	-0.3	13.6	11.5	even	ok
486.2259	1	C28H33NNaO5	100.00	486.2251	1.6	0.8	5.3	12.5	even	ok
927.4801	1	C56H67N2O10	100.00	927.4790	1.2	1.1	8.4	24.5	even	ok
944.5064	1	C56H70N3O10	100.00	944.5056	-0.9	-0.8	7.8	23.5	even	ok
949.4624	1	C56H66N2NaO10	100.00	949.4610	1.5	1.5	22.1	24.5	even	ok

pangxiaoyan_PH-14_pos_61_01_14942.d
Bruker Compass DataAnalysis 4.1 printed: 4/21/2023 5:10:49 PM by: SCSIO Page 1 of 1

Figure S32. HRESIMS spectrum of **3**.

Table S1. NMR data for compounds **3** and **6** (500/125 MHz, TMS, *δ* ppm) in DMSO-d_6.

Position	**3**		**6**	
	δ_C	δ_H	δ_C	δ_H
1	171.3 C		171.0 C	
2 (NH)		8.35, brs		8.36, brs
3	58.0 CH	3.37, m	58.2 CH	3.39, dd, 10.0, 4.5
4	47.8 CH	3.16, m	47.4 CH	3.28, brs
5	124.3 C		124.2 C	
6	134.9 C		134.1 C	
7	68.9 CH	3.68, t, 8.5	69.1 CH	3.60, brt, 8.5
8	49.2 CH	2.92, brt, 7.0	48.8 CH	2.95-2.98, m
9	84.1 C		84.2 C	
10	42.6 CH_2	2.90, t, 10.0<br>2.60, dd, 13.0, 10.0	42.7 CH_2	2.93, t, 10.0<br>2.68, dd, 13.0, 10.0
11	17.2 CH_3	1.17, s	17.1 CH_3	1.18, s
12	14.8 CH_3	1.50, s	14.5 CH_3	1.51, s
13	127.9 CH	6.06, ddd, 15.0, 10.5, 1.5	127.6 CH	5.81, dd, 15.0, 10.5
14	132.7 CH	5.05, ddd, 13.5, 10.5, 2.5	133.4 CH	5.23, ddd, 15.0, 11.0, 4.0
15	38.4 CH_2	2.26, dt, 13.5, 11.5<br>2.15, dq, 13.5, 2.0	40.2 CH_2	2.21, brd, 14.0<br>1.68-1.77, m
16	42.4 CH	3.07, ddd, 10.5, 6.5, 3.5	39.8 CH	3.47, dqd, 13.0, 6.0, 2.0
17	211.1 C		205.4 C	
18	141.9 C		141.6 C	
19	124.3 CH	5.64, td, 8.5, 1.5	133.0 CH	6.48, dd, 10.0, 6.5
20	32.1 CH_2	3.54, dd, 16.5, 10.5<br>3.10-3.19, m	37.2 CH_2	3.57, t, 10.5<br>2.95-2.99, m
21	170.7 C		168.8 C	
22	18.6 CH_3	1.04, d, 6.5	17.2 CH_3	0.99, d, 6.5
23	19.0 CH_3	1.99, s	12.7 CH_3	1.75, s
1'	137.7 C		137.6 C	
2',6'	129.4 CH	7.16, d, 7.0	128.5 CH	7.17, d, 7.5
3',5'	128.5 CH	7.33, t, 7.5	129.4 CH	7.35, t, 7.5
4'	126.6 CH	7.24, t, 7.5	126.6 CH	7.26, t, 7.5
OH-7		4.64, d, 8.0		4.60, d, 8.0

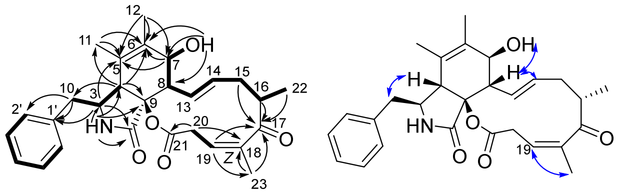

Figure S33. Key ^{1}H-^{1}H COSY (—), HMBC (→), and NOESY (↔) correlations of compound **3**.

ITS1-5.8S-ITS2 sequences of *Aspergillus* sp. SCSIO 41044

TCCGAGGTCAACCTGAAAAAAAGGTGATGCGTGGGCTGGCGCCGGCCGGGCCTACAG
AGCGGGTGACAAAGCCCCATACGCTCGAGGACCGGACGCGGTGCCGCCGCTGCCTTT
CGGGCCCGTCCCCGGGGGGACGAGGACCCAATACAAGCCGGGCTTGAGGGCAGTAAT
GACGCTCGGACAGGCATGCCCCCCGGAATACCAGGGGGCGCAATGTGCGTTCAAAGA
CTCGATGATTCACTGAATTCTGCAATTCACATTAGTTATCGCATTTCGCTGCGTTCTTCAT
CGATGCCGGAACCAAGAGATCCATTGTTGAAAGTTTTAACTGATTACAAAGAATCGGA
TTCATACAGGCTTTCAGAAACAGTGTTCGTGTTGGGGTCTCCGGCGGGCACGGGCCCG
GGGGCAGAAGCCCCCCGGCGGCCAGCAGACGCTGGCGGGCCCGCCGAAGCAACAGT
GGTACAGTAGTCACGGGTGGGAGGTTGGGCCACGAGGACCCTCACTCGGTAATGATCC
TTCCGCAGGTCCCCTTACGGA
